# The freestyle pedicle perforator flap: a new favorite for the reconstruction of moderate-sized defects of the torso and extremities

**DOI:** 10.1007/s00238-014-1043-4

**Published:** 2014-12-21

**Authors:** Gudjon Leifur Gunnarsson, Ian T. Jackson, Tormod S. Westvik, Jorn Bo Thomsen

**Affiliations:** 1 Department of Plastic Surgery, Telemark Hospital, Skien, Norway; 2 Ian Jackson Craniofacial & Cleft Palate Clinic, Beaumont Children’s Hospital, Royal Oak, MI USA; 3 Beaumont Health System Neuroscience Center, Royal Oak, MI USA; 4 Department of Plastic Surgery, Odense University Hospital, Odense, Denmark

**Keywords:** Flap, Perforator, Pedicle, Propeller flaps, Freestyle, Torso, Extremities

## Abstract

**Background:**

Perforating vessels are a consistent anatomical finding and well described in the current literature. Any skin flap can be raised on a subcutaneous pedicle as long as it contains at least one supplying perforator. Perforator flaps have been interlinked with microsurgery and generally not widely performed by the general plastic surgeons. The aim of this paper is to present the simplicity of pedicled perforator flap reconstruction of moderate-sized defects of the extremities and torso.

**Methods:**

We retrospectively reviewed the charts of 34 patients reconstructed using 34 freestyle pedicled perforator flaps for moderate-sized defects of the truncus and extremities. We registered indications, flap size and localization, success rate, and complications. Most importantly, we describe a simple approach to the design of freestyle pedicled perforator flaps and elaborate on technical aspects in the context of current literature.

**Results:**

The reconstructive goals were achieved in all cases without any total flap loss or major complications. Minor complications occurred in 7/34 (21 %) cases consisting of venous congestion leading to distal tip necrosis or epidermolysis; partial flap loss was significant in 4 cases, however never more than 10 % of the total flap size. Reconstruction was performed on the lower limb in 13 cases, upper limb in 12, and 9 cases were on the truncus. The angle of rotation was 90° in 21 cases and 180° in 13 cases. The most common indication was reconstruction of oncological skin defects; melanoma 19, BCC 6, SCC 2, other 7. The flap size varied from 1.5×3 cm to 12×22 cm. The perforator identification was done by intraoperative exploration in 17 cases and by color Doppler ultrasonography in 17 cases.

**Conclusions:**

Moderate-sized defects of the torso and extremities can be successfully reconstructed by pedicled perforator flaps. The flap dissection is simple, and the complication rates comparable to other reconstructive options.

Level of evidence IV, therapeutic study

## Introduction

The evolution of modern flap surgery and microsurgery is interlinked with the understanding of skin flap anatomy and clinical observations. The presentation of the axial pattern flap by Jackson and McGregor in the 1970s, [1, 2] marked the beginning of a decade of fasciocutaneous and myocutaneous flaps in 1980s, followed by a more profound use of perforator flaps in 1990s [3, 4]. Ian Taylor and associates have shown that skin vascularity is known to be supplied by perforators in a persistent anatomical pattern that applies directly to their use in flap surgery [5]. The angiosome originally described by Taylor and Palmer [6] in 1987 has been further narrowed down to a perforasome to explain the reliable area of skin supplied by a single perforator [7]. One major challenge in the application of the perforator flap principle is the establishment of a safe flap territory. Knowing the safe boundaries of the perforasome will most certainly result in a wider range of application. The safety of the freestyle perforator flap for the reconstruction of a medium-sized defect in the face has been established [8]. The aim of this paper is to present the simplicity of pedicled perforator flap harvest and reconstruction of the moderate-sized defects of the extremities and torso using freestyle pedicle perforator flaps.

## Material and methods

We performed a retrospective study based on 34 consecutive patients, 16 men and 18 women aged 37–93, who had moderate-sized defects on the torso and limbs reconstructed using freestyle pedicled perforator flaps. Two similar operative methods, termed A and B, were used differing only in the way perforators were identified. In half of the patients (*n* = 17), the perforator was identified preoperatively using color Doppler ultrasonography (CDU) and the other half (*n* = 17) by intraoperative exploration alone.

### Operative technique A and B

#### Preoperative color Doppler ultrasonography identification (Fig. 1)

**Fig. 1:**
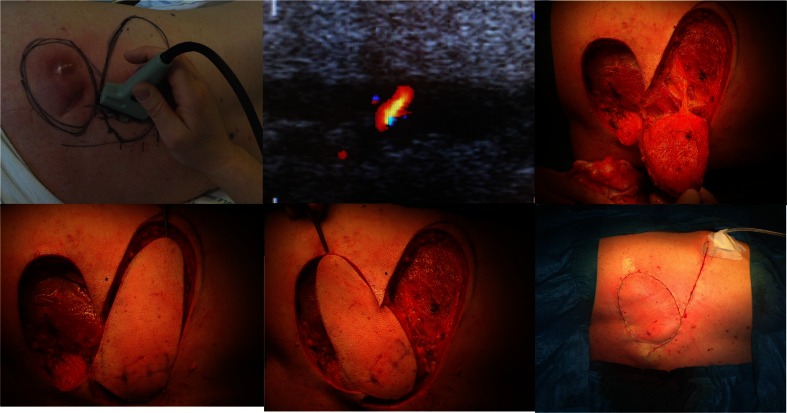
Operative technique demonstrating the CDU-assisted freestyle technique. A sizeable perforator is localized at the base of the planned flap along the rotation axis

We used a BK Medical color Doppler ultrasonographer with a 10–12 mHz linear transducer. The settings were set for small peripheral vessels and low flow velocity to enable detection of flow in the perforators. Skin availability adjacent to the defect was evaluated in terms of possible flap options. Once identified, the area of interest was examined moving the transducer very slowly until a pulsating perforator was identified at the deep fascia level. Perforators in the area were mapped according to size and located with a permanent marker. The skin flap was subsequently designed to include the biggest perforator available and preferably to allow the least possible angle of rotation including as many perforators as possible in the design.

#### Intraoperative exploration (Fig. 2)

**Fig 2:**
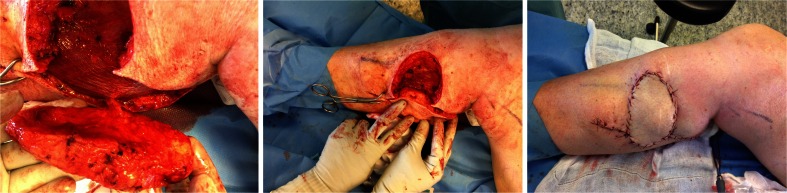
Intraoperative exploration. The defect is extended in a subfascial level until a suitable perforator is found and then the flap is designed around it

The wound edges of the existing defect sufficiently allow for exploration at the level of the deep fascia to locate a suitable perforator to supply a nearby local flap.

The final flap design was based on the perforator location and available tissue. When a suitable perforator was localized, the flap design of a transposition or propeller flap was completed in the shape of the defect and preferably including the donor site as well. The flap was dissected of the underlying muscle fascia and released circumferentially as an island until the necessary range of movement was obtained.

In most cases, the perforators were not skeletalized, and a small cuff of subcutaneous tissue surrounding the pedicle was preferably preserved. Perforators were not dissected beyond the level of the muscle fascia.

The flaps were inset with a combination of a resorbable polyglactine and nylon suture, and the donor site closed primarily in a linear fashion. Drains were not routinely used. Light dressings were applied, and the patient was discharged at the day of surgery or the following day depending on the indication and general medical condition.

## Results

Moderate-sized defects of the torso and limbs were reconstructed successfully using a freestyle pedicled perforator flap in 34/34 (100 %) cases. We did not experience any total flap loss or major complications; however, minor complications were registered in 7/34 (21 %); congestion, epidermolysis, and peripheral partial flap necrosis of less than 10 % total flap size. The complications occurred in the lower limbs in 4/7 cases and distal upper limbs in 3/7. In 6/7 of the cases with complications, the arc of rotation of the propeller flap was 180°. The donor sites were closed directly in all cases. The defect location was distributed evenly; 13 lower limb, 11 upper limb, and 10 torso. The most frequent indication for surgery was tumor resection 26/34 (70 %), followed by scar correction 6 and chronic wound or trauma in 4 cases, Table 1. Flap rotation ranged from 90 to 180°. One hundred eighty degrees in 12/34 (35 %) and 90° in 22/34 (65 %).

**Table 1:** Patient data and outcome

FN	A/S	I	DL/FL	FT	PI	RO	FD	FA	C	R	OC
1	81/F	LMM	UL	P	FS	180	3 × 7	21	N	N	RA
2	80/M	MM	UL	P	FS	90	4 × 7	28	N	N	RA
3	63/M	MM	UL	P	FS	180	8 × 12	96	MN	N	RA
4	76/F	EB	UL	P	FS	180	6 × 17	102	N	N	RA
5	53/M	TR	UL	P	FS	180	2 × 6	12	N	N	RA
6	76/M	MM	UL	P	FS	90	4 × 7	28	N	N	RA
7	57/M	KS	UL	P	FS	90	7 × 16	112	N	N	RA
8	82/F	MM	UL	P	CDU	180	5 × 13	65	MN	N	RA
9	67/M	MM	UL	P	CDU	180	5 × 9	45	MN	N	RA
10	59/M	MM	UL	P	CDU	90	5 × 8	40	N	N	RA
11	59/M	MM	UL	P	FS	90	9 × 12	108	N	N	RA
12	64/F	MM	LL	P	FS	180	4 × 10	40	MN	N	RA
13	64/F	MM	LL	P	FS	90	7 × 12	84	MN	N	RA
14	93/M	BCC	LL	P	FS	90	6 × 9	54	N	N	RA
15	55/F	MM	LL	P	FS	180	1,5 × 3	4, 5	N	N	RA
16	72/F	EKP	LL	P	FS	90	12 × 22	264	N	N	RA
17	57/M	MM	LL	P	CDU	90	5 × 9	45	N	N	RA
18	67/M	BCC	LL	P	CDU	90	4 × 7	28	N	N	RA
19	88/F	BCC	LL	P	CDU	90	5 × 8	40	N	N	RA
20	63/F	SCC	LL	P	CDU	90	2,5 × 5	12,5	N	N	RA
21	55/F	MM	LL	P	CDU	180	4 × 8	32	MN	N	RA
22	64/F	SCC	LL	P	CDU	90	4 × 8	32	N	N	RA
23	37/F	MM	LL	P	CDU	180	4 × 6	24	N	N	RA
24	50/F	MM	LL	P	CDU	90	4 × 12	48	MN	N	RA
25	63/M	MM	B	P	FS	180	7 × 20	140	N	N	RA
26	71/M	KS	B	P	FS	90	6 × 7	42	N	N	RA
27	44/F	BCS	B	P	FS	90	4 × 10	40	N	N	RA
28	54/F	BCS	B	P	FS	180	7 × 22	154	N	N	RA
29	65/M	BCC	B	P	CDU	90	7 × 17	117	N	N	RA
30	59/F	MM	B	P	CDU	90	8 × 21	168	N	N	RA
31	63/M	MM	B	P	CDU	90	4 × 10	40	N	N	RA
32	73/M	CW	B	P	CDU	90	8 × 12	128	N	N	RA
33	68/F	BCS	B	P	CDU	90	5 × 19	95	N	N	RA
34	53/F	BCS	B	P	CDU	90	7 × 13	91	N	N	RA

*FN* flap number, *A/* age/sex patient, *I* indication, *DL/FL* defect/flap location, *FT* flap type, *PI* perforator identification, *RO* rotation in degrees, *FD* flap dimensions, *FA* flap area, *C* complications, *R* revision, *OC* outcome, *LMM* lentigo malignant melanoma, *UL* upper limb, *P* propeller, *FS* freestyle, *N* no, *RA* reconstruction achieved, *MM* malignant melanoma, *VC* venous congestion, *SS* secondary suture, *EB* exposed bone, *TR* Trauma, *EKP* exposed knee prosthesis, *LL* lower leg, *MN* marginal necrosis (less t. 10 %), *SH* secondary healing, *BCC* basal cell carcinoma, *KS* keloid/Scarring, *B* body, *BCS* breast conserving surgery, *CDU* color Doppler ultrasonography. *CW* Chronic wound *DD* donor defect dehiscence

## Discussion

**Fig 3:**
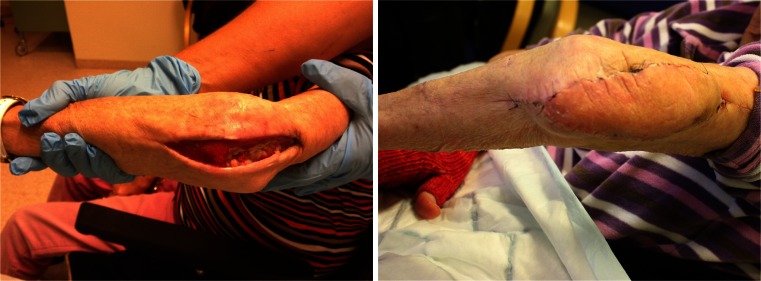
An example of a “ propeller” design flap for a chronically exposed elbow fracture

**Fig 4:**
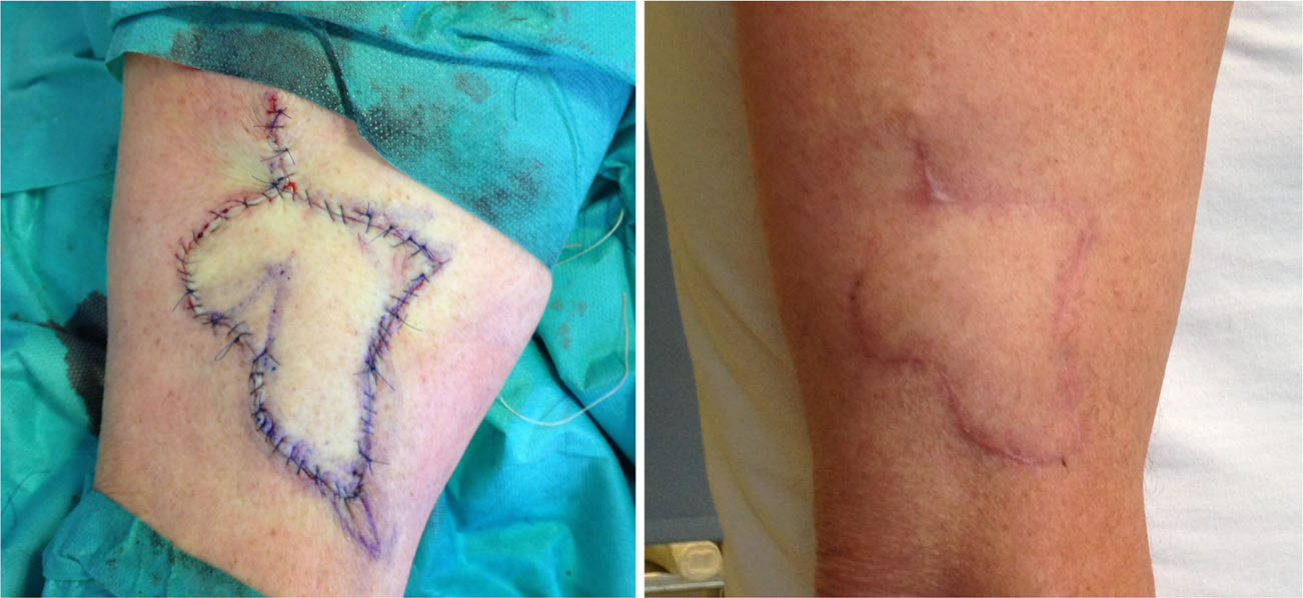
An example of the multilobular “cogwheel” design

**Fig 5:**
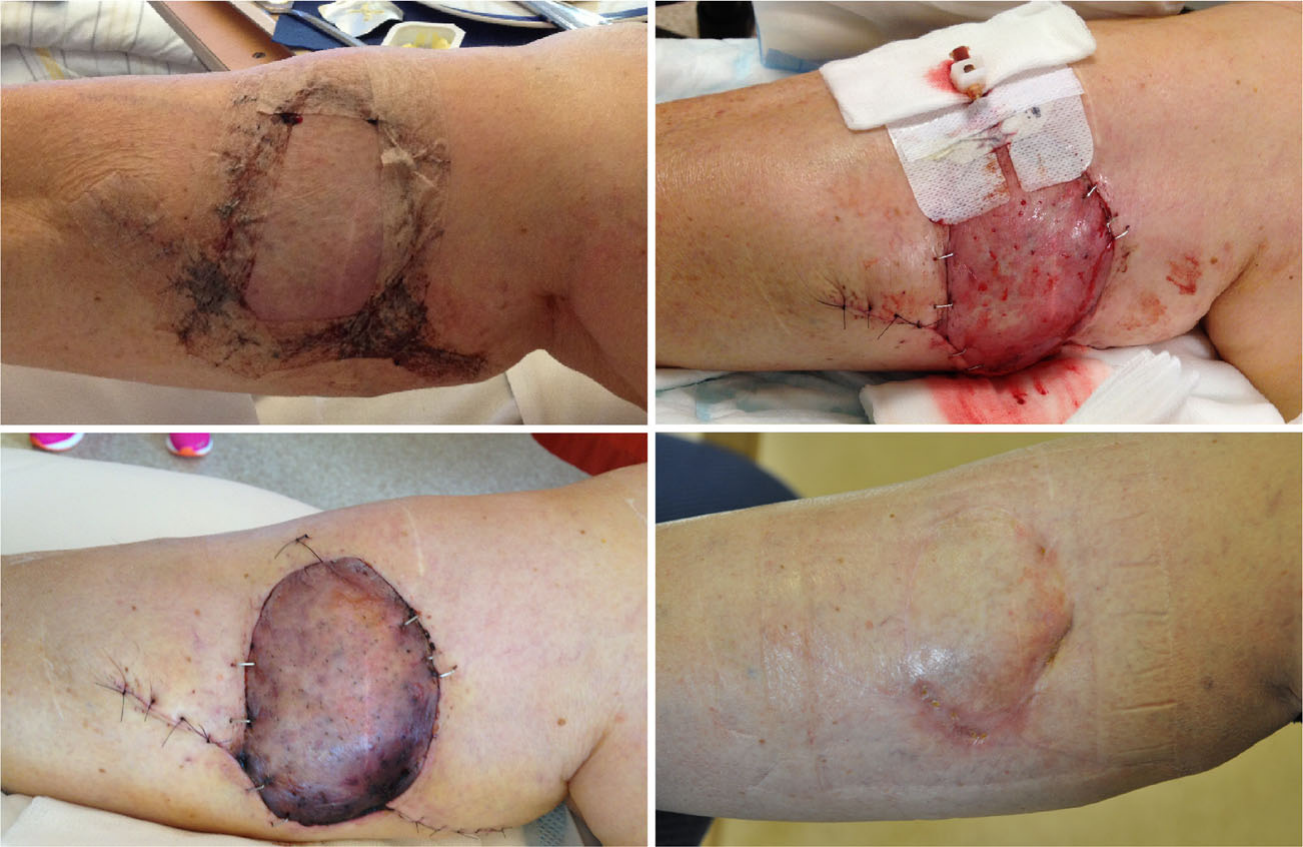
At the day of discharge, the patient was mobilized and the flap became congested. A bedside fenestration was attempted for salvage. The case shows a typical resolution in the case of congestion and partial necrosis without operative revision

The freestyle flap concept was first introduced in 1983 by the Finnish surgeon Asko-Seljavaara [9]. It refers to the wide array of perforators that can safely supply a skin flap within the given perforasome [7]. The perforator located nearest to the defect allows for the shortest angle of rotation and simplest flap design. The pedicled perforator flap is a good option for reconstruction of the moderate-sized defect as it is most likely within the safe boundaries of the chosen perforasome and the donor site can be closed directly. The perforators were consistently found where to be expected by anatomical description [6, 7] and readily localized with CDU. When the perforator was localized, the flap was simply released to allow for the necessary range of rotation without any tension. As opposed to most published series, the perforator was not dissected down to the axial vessel [10–12]. We have used several different flap designs, ranging from a simple ellipse (Fig. 3) to a multilobular flap, which we call a “cogwheel” flap (Fig. 4). Our experience so far indicates that the technique is simple and reliable despite a limited sample size. Complications encountered were of minor character consisting of venous stasis and subsequent epidermolysis or partial necrosis that healed by secondary intention without further management (Fig. 5). Consistent with previous reports, flap congestion was more pronounced in the lower limb [12–14] and distal forearm [15, 16]. The effect of a potential torsion of a short pedicle occurring when the flaps were rotated 180° on the limbs may be a factor in venous congestion, but this series is too small conclude on it and more factors are most likely involved such as the effect of gravity and insufficient venous drainage [17]. The incidence of complications is largely unknown and varies by reports, highest for the lower limb, ranging from 17.2–25.8 % with venous congestion and partial flap necrosis being most frequently reported and total flap loss ranging from 1.1–5.6 % [12–14]. The incidence of complications in the body and upper limb is somewhat less well documented ranging from 19.3 to 23 % [15, 16]. This shows that complications are often encountered. Despite the alarming numbers, most are minor and require just a simple solution no more extensive than the routine care of a split thickness skin graft donor site. The increased use of freestyle pedicled perforator flaps has markedly reduced our need for skin grafts and has become our first choice of primary as well as secondary reconstruction of moderate-sized defects.

## Conclusion

The freestyle pedicled perforator flap is a safe reconstructive option for the moderate-sized defect of the truncus and extremities. Perforators are consistently found where to be expected and can be readily localized using a preoperative CDU or by intraoperative exploration. Flap harvesting is simple to perform with satisfying results, a reasonable margin of safety, and a forgiving donor site that makes it ideal for the initial practice of perforator flaps. Complications occur most commonly in the distal lower leg and distal forearm and less on the torso. In our practice, these flaps have become the standard of care for moderate-sized defects too large for direct primary linear closure.

## References

[1] McGregor IA, Jackson IT (1972). The groin flap. Br J Plast Surg.

[2] Smith PJ, Foley B, McGregor IA, Jackson IT (1972). The anatomical basis of the groin flap. Plast Reconstr Surg.

[3] Kroll SS, Rosenfield L (1988). Perforator-based flaps for low posterior midline defects. Plast Reconstr Surg.

[4] Koshima I, Soeda S (1989). Inferior epigastric artery skin flap without rectus abdominis muscle. Br J Plast Surg.

[5] Taylor GI (2003). The angiosomes of the body and their supply to perforator flaps. Clin Plast Surg.

[6] Taylor GI, Palmer JH (1987). The vascular territories (angiosomes) of the body: experimental study and clinical applications. Br J Plast Surg.

[7] Saint-Cyr M, Wong C, Schaverien M, Mojallal A, Rohrich RJ (2009). The perforasome theory: vascular anatomy and clinical implications. Plast Reconstr Surg.

[8] Gunnarsson GL, Jackson IT, Thomsen JB (2014). Freestyle facial perforator flaps—a safe reconstructive option for moderate-sized facial defects. Eur J Plast Surg.

[9] Asko Seljavaara S.(1983) In: Abstracts of the Seventh Congress of the International Society of Reconstructive Microsurgery; June 19–30, 1983; New York, NY

[10] Lecours C, Saint-Cyr M, Wong C, Bernier C, Mailhot E, Tardif M, Chollet A (2010). Freestyle pedicle perforator flaps: clinical results and vascular anatomy. Plast Reconstr Surg.

[11] D’Arpa S, Cordova A, Pignatti M, Moschella F (2011). Freestyle pedicled perforator flaps: safety, prevention of complications, and management based on 85 consecutive cases. Plast Reconstr Surg.

[12] Gir P, Cheng A, Oni G, Mojallal A, Saint-Cyr M (2012). Pedicled-perforator (propeller) flaps in lower extremity defects: a systematic review. J Reconstr Microsurg.

[13] Nelson JA, Fischer JP, Brazio PS, Kovach SJ, Rosson GD, Rad AN (2013). A review of propeller flaps for distal lower extremity soft tissue reconstruction: is flap loss too high?. Microsurg.

[14] Menichini G, Baldrighi C, Delcroix L, Vignini L, Tos P (2014). Are there risk factors for complications of perforator-based propeller flaps for lower-extremity reconstruction. Clin Orthop Relat Res.

[15] Lazzeri D, Huemer GM, Nicoli F, Larcher L, Dashti T, Grassetti L, Li Q, Zhang Y, Spinelli G, Agostini T (2013). Indications, outcomes, and complications of pedicled propeller perforator flaps for upper body defects: a systematic review. Arch Plast Surg..

[16] Innocenti M, Baldrighi C, Delcroix L, Adani R (2009). Local perforator flaps in soft tissue reconstruction of the upper limb. Handchir Mikrochir Plast Chir.

[17] Pribaz JJ, Chan RK (2010). Where do perforator flaps fit in our armamentarium. Clin Plast Surg.

